# The Effect of Bio-Based Polyamide 10.10 and Treated Fly Ash on Glass-Fiber-Reinforced Polyamide 6 Properties

**DOI:** 10.3390/polym17141950

**Published:** 2025-07-16

**Authors:** George-Mihail Teodorescu, Zina Vuluga, Toma Fistoș, Sofia Slămnoiu-Teodorescu, Jenica Paceagiu, Cristian-Andi Nicolae, Augusta Raluca Gabor, Marius Ghiurea, Cătălina Gîfu, Rodica Mariana Ion

**Affiliations:** 1 National Institute for Research & Development in Chemistry and Petrochemistry—ICECHIM, 202 Splaiul Independentei, 060021 Bucharest, Romania; toma.fistos@icechim.ro (T.F.); cristian.nicolae@icechim.ro (C.-A.N.); raluca.gabor@icechim.ro (A.R.G.); marius.ghiurea@icechim.ro (M.G.); catalina.gifu@icechim.ro (C.G.); rodica.ion@valahia.ro (R.M.I.); 2 Doctoral School of Materials Engineering Department, “Valahia” University of Targoviste, 13 Aleea Sinaia, 130004 Targoviste, Romania; 3 Materials Engineering and Mechanics Department, “Valahia” University of Targoviste, 13 Aleea Sinaia, 130004 Targoviste, Romania; sofiateodorescu@yahoo.com; 4 CEPROCIM S.A., 6 Preciziei, 062203 Bucharest, Romania; jenica.paceagiu@ceprocim.ro

**Keywords:** polyamide 6, bio-based polyamide 10.10, fly ash, glass fiber, composites

## Abstract

Increased concern for human health and the environment has pushed various industries to adopt new approaches towards satisfying modern regulations. Strategies to achieve these approaches include utilizing lightweight materials, repurposing waste materials, and substituting synthetic polymers with bio-based counterparts. This study investigates the effects of treated fly ash (C) and bio-based polyamide 10.10 (PA10) on the thermal, morphological, and mechanical properties of glass fiber (GF)-reinforced polyamide 6 (PA6). Our main objective was to develop a composite that would allow for the partial replacement of glass fiber in reinforced polyamide 6 composites (PA6-30G) while maintaining a favorable balance of mechanical properties. Composites processed via melt processing demonstrated enhanced mechanical properties compared to PA6-30G. Notably, significant improvements were observed in impact strength and tensile strain at break. The addition of PA10 resulted in increases of 18% in impact strength and 35% in tensile strain relative to PA6-30G. Complementary, structural and morphological analyses confirmed strong interfacial interactions within the composite matrix. These findings indicate that a PA6/PA10 hybrid composite may represent a viable alternative material for potential automotive applications.

## 1. Introduction

Petroleum-based polymers have been used for many years in various industries; however, due to their resistance to various kinds of biological and chemical degradation, once they are thrown out in the environment, they exhibit a prolonged and serious risk towards nature and humans alike [1,2]. Therefore, researchers are pushing for advances in polymer biocomposites with improved performance and prices that are at least comparable to synthetic polymer composites. For these biocomposites to perform at a similar or improved level compared to petroleum-based composites, they are commonly reinforced with materials like glass fibers, carbon nanotubes, clays, silica, and graphite–all of which are completely non-biodegradable, inorganic, and are often derived from petroleum [3]. In the automotive industry, innovation follows a similar trend with the replacement of conventional synthetic polymer composites with eco-friendly alternatives. A particular focus has been put on the processing and characterization of eco-friendly composites, particularly material categories such as thermosetting and thermoplastic, nanocomposites, and layered and polymer biofoams for use in the automotive industry [4]. Another current trend in the automotive industry is the employment of lightweight materials that are focused on the reduction and replacement of glass fiber content in car parts with more cost-effective reinforcement agents without greatly reducing the overall mechanical properties of the part.

Glass-fiber-reinforced polymer composites (GFCs), notably polyamide 6 (PA6) reinforced with glass fiber, are widely used in automotive applications. These composites offer superior strength, stiffness, and durability compared to neat PA6 due to effective stress transfer across the matrix/fiber interphase [5,6,7]. This versatile composite can also be easily modified to improve its mechanical properties, such as its strength and stiffness, which makes it ideal for use in applications that require high load-bearing capacity and durability, and will continue to be a go-to choice for many applications. A significant downside of glass fiber in polymer composites is, however, the high-loading requirements needed to reach its desired characteristics, along with its recycling complications. In light of this, researchers explore natural-fiber-reinforced composites (NFCs) as partial or full substitutes for glass fiber to reduce environmental impacts and energy consumption [8,9]. Studies indicate that NFCs can achieve mechanical properties comparable to GFCs (e.g., modulus, tensile strength) in specific applications while offering environmental advantages like lower weight and improved recyclability [10,11,12]. Key limitations of NFCs include the high required loadings of natural fibers (potentially exceeding glass fiber) and the requirement for fiber treatment before high-temperature processing, as natural fibers tend to degrade at the processing temperature of a polymer such as polyamide 6.

An alternative lightweight strategy involves utilizing cost-effective reinforcements like aluminosilicate-rich fly ash waste. Recent studies demonstrate that incorporating fly ash into glass-fiber-reinforced polypropylene enhances its mechanical properties via strong polymer–filler interactions [13,14]. Other studies have investigated the effect of natural fillers, resulting from renewable resources, such as by-products or industrial waste (cellulose fibers, peanut shell powder, olive pomace powder, olive stone powder, or rice husk ash), on the mechanical and biodegradability properties of PA6 and fully or partially bio-based polyamides such as PA10, PA6.10, and PA5.6 [15,16,17,18]. These composites, which were obtained either by melt-compounding or directly through a synthesis process, presented enhanced mechanical properties compared to neat polymers with improvements, especially in rigidity (when using rice husk ash with or without clay addition in PA6.10 and PA10) and impact strength (with cellulose fibers), and enhancement regarding biodegradation with the incorporation of peanut shell powder in PA6 or olive stone powder in PA5.6. Composites based on PA6.10 and PA10.10 reinforced with regenerated cellulose fibers exhibited much-improved toughness properties (impact strength and tensile strain), at the expense of strength and stiffness properties, compared to glass-fiber-reinforced composites.

As mentioned above, the promotion of biocomposites (biodegradable polymers or bio-based materials) is ever-increasing, with more new studies regarding these materials being publicized every year. Bio-based polyamide 10.10 (PA10) exhibits promising mechanical properties, but faces cost barriers for large-scale glass-fiber-reinforced polymer composites production [4]. There are very few studies on this emerging polymer, and the existing ones have applications in various domains such as meat preservation, structure applications, fire resistance behavior, and biogenic wood-plastic composites [19,20,21,22]. A cost-effective approach to using PA10 in automotive applications may involve the melt-mixing of PA6 and PA10, where the resulting blend shares the properties of both polymer matrices.

To obtain a homogeneous mixture, dispersion and compatibility agents are usually required, particularly when working with materials that contain weak polar reactive groups. Commercial macromolecular compatibilizers, which are polyolefins treated with maleic anhydride, are commonly used to improve the interfacial adhesion between polymers and fillers. For polyamides, a maleic anhydride-grafted ethylene copolymer with a low glass transition temperature (T_g_) is mainly used for polymer modification, as shown in a study by Cogen et al. [23]. As for dispersion agents, materials such as poly(propylene glycol adipate) may be used to treat the surface of fillers to enhance their dispersion in the polymer matrix [13,24]. This dispersion agent has been demonstrated to function as a plasticizer and enhance the compatibility between the composite components and the general mechanical properties. Alkoxysilanes such as 3-(glycidyloxypropyl) trimethoxysilane can also be used to treat the surface of nano-silica [25] in order to achieve a better dispersion of particles within a polymer matrix. In a recent study [26], the surface of mesoscopic fly ash was grafted with 3-(glycidyloxypropyl) trimethoxysilane as a coupling agent to improve the mechanical properties of the studied composites.

To our knowledge, there have been no studies that investigate the interaction between polyamide 6, polyamide 10.10, glass fiber, and fly ash. As such, the current study can provide a basis for future studies to obtain and characterize glass-fiber/fly-ash-reinforced polyamides. The novelty of this article consists of the use of a specific type of fly ash waste and a bio-based polyamide, along with two agents for the surface treatment of fly ash, that are rarely used in such compositions, and studying the effect of each component on the properties of the final composite and the interactions between them.

The objective of this study is to investigate glass-fiber-reinforced polyamide 6, bio-based polyamide 10.10, and treated fly ash waste composites with the purpose of obtaining a eco-friendly and cheaper alternative for partial replacement of glass fiber and obtain new composites with a good balance of mechanical properties and that are easier to biointegrate at the end of the life cycle. A percentage of glass fiber will be replaced with fly ash, and bio-based polyamide 10.10 will be added to improve the strain at break (%) of the composite. The final composition will be compared to the commercial 30% wt. glass-fiber-reinforced polyamide 6 composites used in the automotive industry. This reduction in glass fiber must not compromise the essential properties required for automotive materials.

## 2. Materials and Methods

### 2.1. Materials

A commercial polyamide 6 reinforced with 30% glass fiber (GF) (SAXAMID 126F6, SAX Polymers, Vienna, Austria), with a tensile elongation at break of 4%, marked with PA6-30G, was used as the polymer matrix. Commercial polyamide 6 (SAXAMID 126N, SAX Polymers, Vienna, Austria), marked with PA6, was used to dilute the PA6-30G to the desired fiber–glass concentration. A bio-based polyamide 10.10 (NP BioPA 1010-201, Nature Plast, Mondeville, France) with a tensile elongation at break >50%, was abbreviated with PA10 and was added to improve the elasticity (strain at break %) of PA6-30G. Fly ash powder (C), resulting from the process of obtaining basalt wool (from the electrofilter) was used as a reinforcing filler and was sourced from a thermal power plant located in Govora (CET Govora S.A., Râmnicu Vâlcea, Romania). Poly(propylene glycol adipate) (Solventul S.A., Timisoara, Romania) was marked with P and used as a dispersion agent for fly ash as well as 3-(glycidyloxypropyl) trimethoxysilane (Sigma Aldrich, Saint Louis, MO, USA), which was marked with S and used with the same purpose. A maleic anhydride grafted ethylene copolymer, mainly used for polymer modification, especially in polyamide toughening, Fusabond N493 (Dupont, Wilmington, DE, USA), was used as a compatibilizing agent.

### 2.2. Preparation of Composites

In a previous article by Teodorescu et al. [13], a detailed discussion regarding the treatment with a dispersion agent and preparation of fly ash as a reinforcing filler in polymer composites was made. Drying, sieving, and milling of ash powder to particle sizes < 90 µm and moisture content < 0.1% was achieved. Furthermore, the oxidic chemical composition for this ash was determined, and it revealed three major components: SiO_2_ (40.19%), K_2_O (18.05%), and Na_2_O (8.92%). Poly(propylene glycol adipate) was used to treat the surface of fly ash, and the modified fly ash (marked with CP) was characterized using X-ray diffraction, FTIR, and SEM analysis [13]. In the same way, the surface of the fly ash was also treated with 3-(glycidyloxypropyl) trimethoxysilane, with the modified fly ash being denoted by CS. The treated ash powder (CP and CS), PA6, PA10, and compatibilizing agent were mixed and dispersed within the PA6-30G matrix using a Leistritz LSM 30.34 co-rotating twin-screw extruder (Leistritz Extrusionstechnik GmbH, Nürnberg, Bayern, Germany) at temperatures between 210 and 240 °C (from hopper to die), with a screw rotation speed of 120 rpm and a feeding rate of 80 rpm. The produced filaments were then cooled and granulated using a Leistritz Granulator system (Leistritz Extrusionstechnik GmbH, Nürnberg, Bayern, Germany). Standard specimens for impact and tensile tests were obtained by injection molding using an Engel VC 60/28 (Engel, Schwertberg, Austria) at an injection speed of 2 mm/min, a temperature range of 220–240 °C, a mold temperature of 80 °C, and a pressure of 600 kg/cm^2^. The final composite formulations are detailed in Table 1.

### 2.3. Characterization

#### 2.3.1. X-Ray Diffraction

CuK_α1_ radiation (λ = 1.5406 Å) was used to collect X-ray diffraction data using the Rigaku Smartlab diffractometer (Rigaku Corporation, Tokyo, Japan). The emission current was kept constant at 200 mA, while the generator radiation acceleration voltage was adjusted to 45 kV during the testing. At room temperature, diffractograms were collected using parallel beam geometry, scanning at 2θ = 5° to 55° at a rate of 4°/min in 0.02° continuous increments. To obtain the interplanar distance (d) of PA in composites, Bragg’s equation was used:

(1) nλ=2d sinθ

where the diffraction angle (θ), the wavelength (λ) of the X-ray, the distance between the crystal lattice planes that create the diffraction indicated asd, and the order of reflection (n) are all represented in this equation. A ±0.05% error margin was maintained.

Using Rigaku PDXL 2 software, crystallite sizes, intensity values, heights, and FWHM (full width at half-maximum intensity of the diffraction peak) values were obtained. The Powder Diffraction File^TM^ (PDF) from the International Centre for Diffraction Data (ICDD) was used to identify crystalline phases. Diffraction profiles were fitted with curves to isolate individual peaks.

#### 2.3.2. Fourier-Transform Infrared Spectroscopy (FTIR)

The FTIR spectra were obtained using a Bruker Vertex 80 (Bruker Optics GmbH & Co. KG, Ettlingen, Germany) with a diamond ATR module, offering 0.2 cm^−1^ resolution and 0.1% T accuracy. Data were recorded in transmission mode (32 scans) over 4000–400 cm^−1^ without sample preparation. Opus software 7.1 was used for data collection and absorbance vs. wavenumber representation.

#### 2.3.3. Dynamic Light Scattering (DLS)

Zeta potential (ξ) measurements were assessed using dynamic light scattering (DLS) on a Nano Zetasizer ZS (Malvern Instruments, Malvern, UK). Nano Zetasizer ZS instruments calculated zeta potential by determining electrophoretic mobility and applying Henry’s equation. The samples were prepared by dispersing 0.0012 mg of powder in 2 mL of ultrapure water, followed by 2 min of ultrasonication. All measurements were performed at room temperature. Three measurements were performed for each sample with a precision of 0.01 mV.

#### 2.3.4. Thermal Characterization

A TA-Q5000IR thermogravimetric analyzer (TA Instruments, New Castle, DE, USA) conducted the thermogravimetric analysis (TGA). Nitrogen was used as the purge gas at a flow rate of 40 mL/min. Samples weighing 14–16 mg were heated from 30 °C to 700 °C at a rate of 10 °C/min.

A differential scanning calorimeter (DSC) (Q2000, TA Instruments, New Castle, DE, USA) examined the sample’s melting and crystallization properties: normalized enthalpy of melting (ΔH_m_), melting temperature (T_m_), normalized enthalpy of crystallization (ΔH_c_), and crystallization temperature (T_c_). The procedure involved a heat–cool–heat (HCH) cycle in a single run. After a 3 min equilibration at −85 °C, the sample was heated to 240 °C, maintained for two minutes, cooled back to −80 °C for two minutes, and reheated to 240 °C at 10 °C/min with a 25 mL/min flow of 5.0-grade helium. X_c_, the degree of crystallinity, was calculated based on the following formula:

(2) $$X_{c}\left(\%\right)=\frac{{\Delta H}_{m}}{{\left(1-\emptyset \right)\Delta H}_{m}^{o}}\times 100\%$$

where ${\Delta H}_{m}^{o}$ and $\;{\Delta H}_{m}$ represent the heats (J/g) of 100% crystalline PA6 with a value of 230 J/g [27,28] and the melting of PA6 composites, respectively. The weight fraction of the components incorporated in the PA6 matrix is represented by ∅.

#### 2.3.5. Dynamic Mechanical Analysis (DMA)

Using a DMA Q800 (TA Instruments, New Castle, DE, USA), the samples’ storage modulus (E′), loss modulus (E″), and loss factor (tan δ) were calculated in relation to temperature. Using a 20 µm amplitude, 1 Hz frequency, and a 3 °C/min heating rate, samples measuring 35 × 10 × 4 mm (length, width, and thickness) were scanned throughout a temperature range of 30–130 °C.

#### 2.3.6. Mechanical Analysis

The tensile properties of the sample were measured using the Instron 3382 universal testing machine (Instron Corporation, Norwood, MA, USA) following ISO 527 [29]. Seven specimens were tested at 50 mm/min for tensile strength and at 2 mm/min for elastic modulus. The impact strength was assessed with a Zwick HIT5.5 Pendulum Impact Tester (Zwick Roell AG, Ulm, Germany) for seven specimens, applying the Charpy notched impact technique according to ISO 179-1/1 e A [30].

#### 2.3.7. Scanning Electron Microscopy (SEM)

The morphological characteristics of the PA6 and PA6/PA10 hybrid composites were analyzed using a Hitachi TM4000 Plus microscope (Hitachi, Tokyo, Japan) with an accelerating voltage of 15 kV. The tensile test specimen’s active zone was isolated and immersed in liquid nitrogen prior to the SEM investigation. After one minute, the samples were removed, and the specimen-selected zone was broken up into about equal-sized pieces using a pair of pliers. The resultant pieces were subjected to a Q150R Plus (Quorum Technologies, SXE, Lewes, UK) coating of 5 nm gold.

## 3. Results and Discussion

### 3.1. FTIR and DLS Analysis

**Figure 1:**
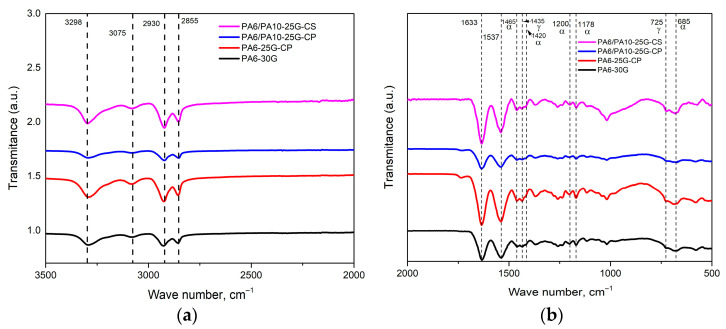
FTIR spectra of PA6-30G, PA6-25G-CP, PA6/PA10-25G-CP, and PA6/PA10-25G-CS composites: (**a**) 3500–2000 cm^−1^ range; (**b**) 2000–500 cm^−1^ range.

The characteristic spectra of PA6 and PA6/PA10 composites are shown in Figure 1. All peaks have been assigned in accordance with previous studies [21,31,32], and the band assignments are given in Table 2. At around 3298 cm^−1^, the stretching vibration of the amine groups (N-H stretching) can be seen, while bands at around 2930 cm^−1^ and 2855 cm^−1^ represent the asymmetric and symmetric stretching vibration peaks of methylene (-CH2-). The band at 3075 cm^−1^ corresponds to the intramolecular bond between amine and carbonyl groups (–CONH2). Around 1633 cm^−1^ and 1537 cm^−1^, the amide I and amide II bands can be recognized, while the peaks at 1465 cm^−1^ and 1435 cm^−1^ represent the C-H asymmetric bending in (-CH2-) or (-CH3-) and (-CH2-) vibration, respectively, and are related to α and γ crystalline phases of PA. The bands at approximately 1200 cm^−1^, 1178 cm^−1^, and 685 cm^−1^, representing different rocking or twisting vibrations of the CH2 and the N–H out-of-plane bending vibration, respectively, are related to the α crystalline form. The peak at about 725 cm^−1^ corresponds to the CH2 rocking modes with four or more C-C rocking vibrations of the bonded atoms and to the N–H out-of-plane bending vibration of the γ form [32,33,34]. The presence of the weak 1420 cm^−1^ peak, related to the α crystalline form and the slight shift and sharpness of the 725 cm^−1^ peak, indicate increased regions of the γ phase [32]. If we consider the maximum intensities of the peaks corresponding to the crystalline phases (α and γ forms) and the ratio between them, we can indirectly have a measure of the content of the alpha and gamma crystalline forms, respectively (α/γ content), as well as the degree of crystallinity. In all the composites studied, the alpha crystalline form predominates. From Figure 1, it can be observed that there is a different variation in the intensity of the peaks in the analyzed samples, most likely caused by interfacial interactions and additive effects which influence the vibrational modes of the functional groups. For example, compared to PA6-30G, for which the α/γ content is 2.42, the higher intensities of peaks in sample PA6-25G-CP may be due to the presence of the polyester diol dispersant with which the fly ash was treated. The polyester diol agent introduces hydroxyl and ester groups, forming strong hydrogen bonds with PA6’s amide groups, which is reflected in the increase in the intensity of the peaks characteristic of amide I and amide II [35,36]. It is also likely that, in this case, the polyester diol agent acts as a bonding promoter, ensuring homogenous fly ash distribution and reducing agglomeration, as was seen in previous studies [13,14]. The increase in the degree of order in the structure is also reflected in the presence of sharper bands. Notable peaks for GF were observed in all samples around 970, 1178, and 1260 cm^−1^, which correspond to Si-OH, Si-O-Si, and the ester carbonyl C=O of silanized GF [37]. The peaks in the region 1100–1000 cm^−1^ that could confirm the presence of treated ash overlap, unfortunately, with the peaks specific to polyamide with glass fiber. The only peak that confirms the presence of treated ash (CP) is the one at 1735 cm^−1^, attributed to the stretching of the ester carbonyl group C=O from polyester diol [13]. However, the treated ash particles may nucleate PA6 crystallization, leading to the sharpening of peaks in the mentioned region, as can be seen in Figure 1b [38]. The PA6-25G-CP composite presents a lower α/γ content, namely 2.15, which means a higher γ phase content. We expect that this increase in the γ crystalline form will be reflected in an increase in toughness, since the γ-form is more ductile than the α-form [39].

In the case of sample PA6/PA10-25G-CP, all peak intensities are lower than all other samples, even compared to PA6-30G. PA10’s lower amide group density and longer methylene chains weaken the H-bond network compared to virgin PA6 [21]. This reduces the intensity of amide peaks (I/II). The lower polarity of PA10 decreases CP-PA6 adhesion. Moreover, the polyester diol plasticizes the matrix, disrupting PA6 crystallinity (lower α phase content) [40]. PA10’s hydrophobicity limits interaction with the hydrophilic polyester diol. This reduces interfacial bonding and overall signal coherence. Compared to PA6-30G, the α/γ content remains unchanged, i.e., 2.42 (at a greater reduction in alpha content compared to gamma), and we expect this behavior to be reflected in a decrease in rigidity in favor of flexibility. For sample PA6/PA10-25G-CS, the higher intensities can be attributed to the influence of the silane agent that forms covalent bonds between itself and fly ash, thus enhancing the signal intensity in the (1000–1100 cm^−1^ region) [41]. A strong increase in the intensity of the peak at 1020 cm^−1^, assigned to Si-O-Si stretching vibrations, is observed, while the peaks at 1045 cm^−1^ and 1080 cm^−1^, assigned to Si-OH groups of fly ash and Si-OC bonds of alkoxy groups, respectively, decrease in intensity, remaining as small shoulders [42]. While PA10’s longer aliphatic chains reduce overall H-bond density, it is possible that the silane agent compensates by improving interfacial adhesion, and this should be reflected in higher stiffness compared to the sample containing CP. For this composite, the α/γ content is 2.26, meaning a slight increase in the content of γ form, which we expect to be reflected in increased toughness.

**Figure 2:**
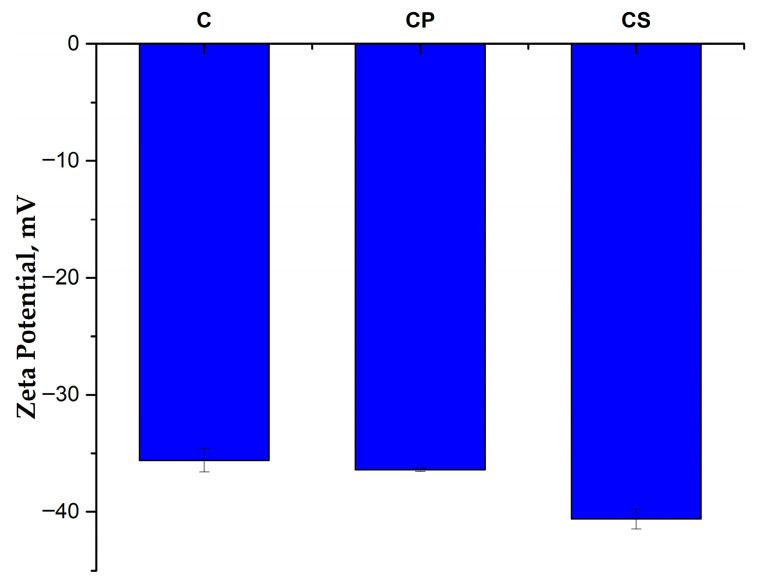
Zeta potential for samples C, CP, and CS.

The FTIR results are also supported by the zeta potential values (Figure 2). The surface-treated ash shows a higher negative zeta potential than the untreated ash (−36.4 mV for CP and −40.6 mV for CS, compared to −35.6 mV for C), which indicates an increase in the electrostatic repulsion between particles, preventing their agglomeration and better dispersion in the polyamide matrix [43,44]. CS shows the highest negative zeta potential value, which suggests a much better dispersion in the polymer matrix and, consequently, improved mechanical properties for the PA6/PA10-25G-CS composite compared to the PA6/PA10-25G-CP composite (both greater stiffness and toughness).

The lack of a visible split in the amide I peak for PA6/PA10-25G-CP and PA6/PA10-25G-CS suggests that there is not any phase separation or immiscibility between PA6 and PA10 [45]. All these results indicate that both γ and α phases are present in the composites, which should be reflected in the mechanical properties of the composites (high toughness and high rigidity).

### 3.2. Differential Scanning Calorimetry Analysis

**Figure 3:**
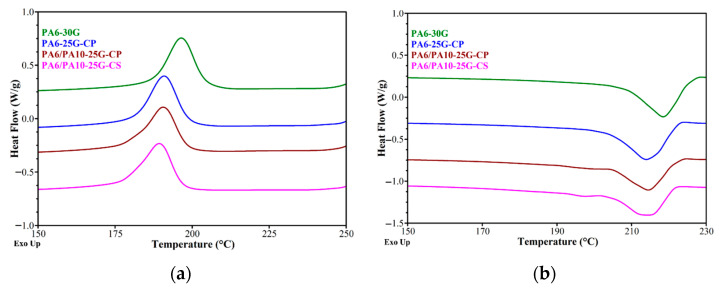
DSC curves of PA6-30G, PA6-25G-CP, PA6/PA10-25G-CP and PA6/PA10-25G-CS composites: (**a**) cooling cycle; (**b**) second heating.

Figure 3 shows the DSC thermograms for PA6 and PA6/PA10 composites reinforced with glass fiber and treated ash powder. In this study, the results from the cooling and second heating are shown and discussed because, as pointed out in a study by Özdilek et al. [46], sample preparation and storage circumstances may have an impact on the first heating findings. The key results specific to the α crystalline phase of PA6, including T_c_ (crystallization exotherm peak), T_m_ (melting endotherm peak), melting/crystallization enthalpy (ΔH_m_/ΔH_c_), and the degree of crystallinity (X_c_) are summarized in Table 3. Similarly to a study performed by Kaßner et al. [47], the crystallinity of PA6-30G in this study is dominated by the α modification. Klata [6] pointed out that β and γ forms of PA6 can reorganize into the α form during DSC scans. The formation of α-phase may also be enhanced due to the slow cooling rate of the samples instead of quenching after the injection molding process [48]. For PA6-30G, a single crystallization peak at 197 °C (Figure 3a) and a melting peak at 218 °C (Figure 3b) are observed, which could be associated with the crystallization and melting of the α crystalline form, respectively.

A study by Blumstein [49] indicated that higher-temperature endotherm may be caused by higher content of fibrillar crystals and higher crystal perfection. Based on the data from Table 3, in the cooling stage a general decrease in enthalpy, onset, and T_c_ can be seen across all samples compared to PA6-30G, most likely because treated fly ash and PA10 disrupt PA6’s crystallization kinetics, and changes in crystal formation occur. It has been reported that an organic compound used for treated the surface of a clay can increase interfacial interactions, but impair the nucleation and crystallization behavior of the polymer [50]. The PA6/PA10-25G-CS composite records the lowest values for the crystallization enthalpy (the lowest number of crystallization nuclei) and for the crystallization temperature (the slowest crystallite growth). Fly ash may act as a nucleating agent, but polyester diol/silane additives likely plasticize the matrix or interfere with chain alignment [51,52]. The similar enthalpy and crystallinity values for PA6-25G-CP and PA6-30G suggest that polyester diol-treated fly ash balances nucleation (improved dispersion) and plasticization (reduced ordering).

For the melting stage, specifically the second melting, decreases in Tm and onset may be caused by the presence of smaller/less perfect PA6 crystals due to filler/polymer interactions. However, compared to PA6-30G, a broadening of the melting range (the difference between the melting and onset temperatures) is observed, which means larger crystals (the largest crystallite size is for PA6-25G-CP). By introducing PA10 into composites based on PA6 with 25% GF and ash treated with polyester diol or silane, it is observed that T_m2_ does not change, but the melting enthalpy and crystallinity decrease by 1–5% and 2–7%, respectively, compared to the PA6-25G-CP composite. In Figure 3b, we notice a melting peak for PA6/PA10-25G-CP and PA6/PA10-25G-CS at around 198 °C that does not appear for PA6-30G and PA6-25G-CP. This peak can be associated with the second melting endotherm peak (the melting temperature of the α crystallites) for PA10, and its position is in accordance with a study performed by Levinta et al. [21].

The DSC results correlate quite well with the FTIR results.

### 3.3. X-Ray Diffraction Analysis

**Figure 4:**
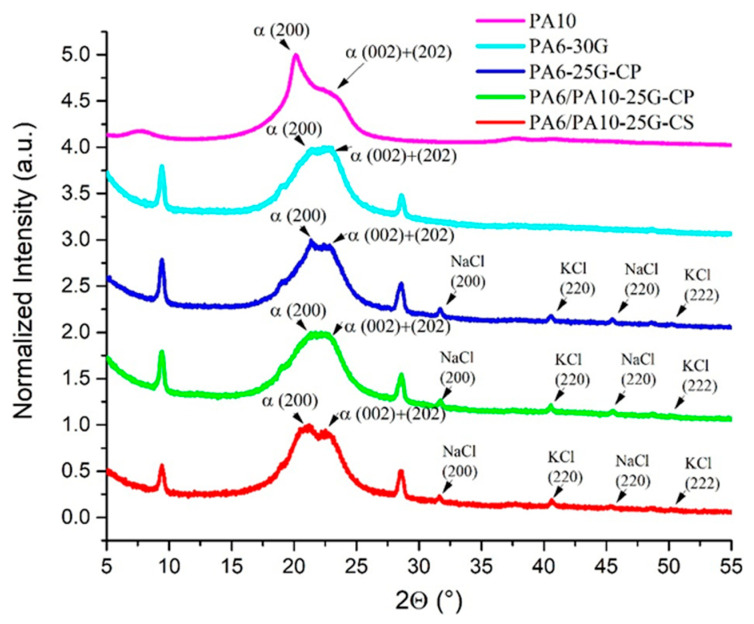
XRD patterns for PA6 and PA6/PA10 composites in comparison to PA6-30G.

XRD patterns for PA6 and PA6/PA10 composites are represented in Figure 4. In studies published by Khanna and Kuhn [53] and by Klata et al. [27], it was noted that a semi-crystalline PA6 can exhibit three main crystallographic forms: a stable α monoclinic form, a metastable pseudohexagonal β form, and an unstable monoclinic γ form. The α form has hydrogen bonds between anti-parallel chains, while the γ form has hydrogen bonds between parallel chains, leading to a twist in the molecular chains in zig-zag planes. The α form is identifiable in Figure 4 through the X-ray diffraction peaks at 2θ = 21.04° and 22.94° associated with the (200) and (002) + (202) crystallographic planes, in accordance with diffraction peaks at 20–21° for PA6 (200) and 23–24° for PA6 (002) + (202) founded by several authors [54,55]. The FTIR results revealed the presence of α and γ crystalline forms. In the X-ray diffractograms, the γ form is not evident. The characteristic peak for the γ crystalline form at approximately 21.5° [56] overlaps with the peaks corresponding to the α form and also with the peak corresponding to talc for (0–21) crystallographic plane, existing in the reference commercial composite (PA6-30G). The addition of treated fly ash can be seen through the presence of several peaks in samples PA6-25G-CP, PA6/PA10-25G-CP, and PA6/PA10-25G-CS. The (200) and (220) NaCl (halite) crystallographic planes (PDF card No. 01-080-3939) were identified at 2θ = 31.69°and at 2θ = 45.42°, respectively, while the (220) and (222) KCl (sylvite) crystallographic planes (PDF card No. 01-076-3378) were identified at 2θ = 28.5°and at 2θ = 40.65°, respectively. The position of these peaks is in accordance with a previous study by Teodorescu et al. [13] that investigated similar polymer composites, confirming the presence of treated and untreated fly ash in the composites based on recycled polypropylene. The characteristic diffraction maxima of talc (Mg_3_(OH)_2_(Si_4_O_10_)) can also be seen at 2θ ≅ 9.42°, 2θ ≅ 19.00°, and 2θ ≅ 28.50° overlapped with that of sylvite for (001), (002), and (003), respectively, crystallographic planes (PDF card No. 01-083-1768). It has been demonstrated that the addition of glass fibers and talc to a polymer matrix can lead to composites with simultaneously improved stiffness and impact resistance. The addition of a single mineral filler (glass fiber or talc) contributes to a substantial improvement in stiffness at the expense of impact resistance [57]. If we consider the peaks corresponding to the crystallographic planes, which do not overlap with the crystallographic planes of other components, namely (001) and (002), it is observed that, compared to the PA6-30G composite, in PA6/PA10-treated fly ash composites, they shift slightly to smaller angles by 0.004–0.006° and by 0.06–0.11°, respectively. This shift means an increase in the d-spacing by approx. 0.06% for the crystallographic plane (001) and by approx. 0.6% for the crystallographic plane (002). At the same time, in PA6/PA10-treated fly ash composites these peaks are narrower by 0.8–1%, indicating larger crystallites. The percentage of peak modification is higher in the case of the PA6/PA10-25G-CS composite, which is evidence of a stronger interaction at the polymer matrix–filler interface. It is possible that the high filler content in the commercial PA6-30G composite suppresses crystallization, thus reducing peak resolution or masking distinct peaks [58,59]. Because of this overlapping, it is difficult to discuss the data resulting from the deconvolution of these peaks.

The diffractograms show, in the range of the amorphous phase signal 2θ =15–28°, a profile composed of wide diffraction maxima of the (200), (002) and (202) diffraction planes of both PA6 and PA10. For sample PA6-25G-CP, the peak corresponding to α (200) diffraction plane shows a shift to the right (higher 2θ) and a lower intensity compared to PA6-30G (Figure 4 and Table 4). This behavior indicates a decrease in d-spacing for the (200) crystallographic plane, possibly due to improved alignment of PA6 chains near the fly ash surface, enhancing their order in specific crystallographic directions. Also, at 2θ = 21.51°, a small sharp peak corresponding to the crystallographic plane (0–21) specific to talc is observed. The α (200 + 202) peak for sample PA6-25G-CP shifts to the left (lower 2θ) and has a lower intensity compared to PA6-30G. This indicates an increase in d-spacing for the overlapping (200 + 202) planes, possibly because of reduced crystallographic coherence, likely due to filler-induced defects, such as fly ash particles acting as heterogeneous nuclei for smaller or misaligned crystallites. It should be noted that these peak shifts might also occur due to residual stress (compressive stress for right shift and tensile stress for left shift) present in the composites [60,61]. The peaks corresponding to the (200), (002), and (202) crystallographic planes become narrower and crystallite size increases. The lower crystallinity in PA6-25G-CP compared to PA6-30G reflects disrupted crystallization kinetics caused by the treated fly ash.

The presence of PA10 in the PA6/PA10-25G-CP composite is reflected in the shift in the peak corresponding to the diffraction plane (200) to lower angles, the broadening (by approx. 22%), and the decrease in peak intensity (by approx. 3%), compared to the PA6-25G-CP composite. The peak corresponding to the (002) and (202) diffraction planes decreases significantly in intensity (by approx. 44%), which is evidence of disorder in the structure as a result of the disrupting PA6 crystallinity by PA10 (Table 4). The diffraction peaks for PA6/PA10-25G-CS are better differentiated due to larger crystalline domains than those of PA6/PA10-25G-CP, which should be reflected in an increase in rigidity of the composites. The peaks corresponding to the diffraction plane (200) shift to lower angles, increases significantly in intensity (by approx. 22%) and narrows by approx. 11% (crystallite size increases), compared to the PA6/PA10-25G-CP composite, highlighting the increase in order in the structure and increase in crystallinity. The peak corresponding to the crystallographic planes (002) and (202) increases less in intensity (by approx. 9%) and broads by approx. 18%, which is evidence the formation of smaller crystallites compared to the PA6/PA10-25G-CP composite. We expect this behavior to be reflected in improved properties for the PA6/PA10-25G-CS composite. The undifferentiated overlap of the maxima in PA6/PA10-25G-CP may be due to a lower degree of organization of the amide chains between PA6 and PA10 or due to some tensioned structures on the surface of the samples [55].

### 3.4. Thermogravimetric Analysis

**Figure 5:**
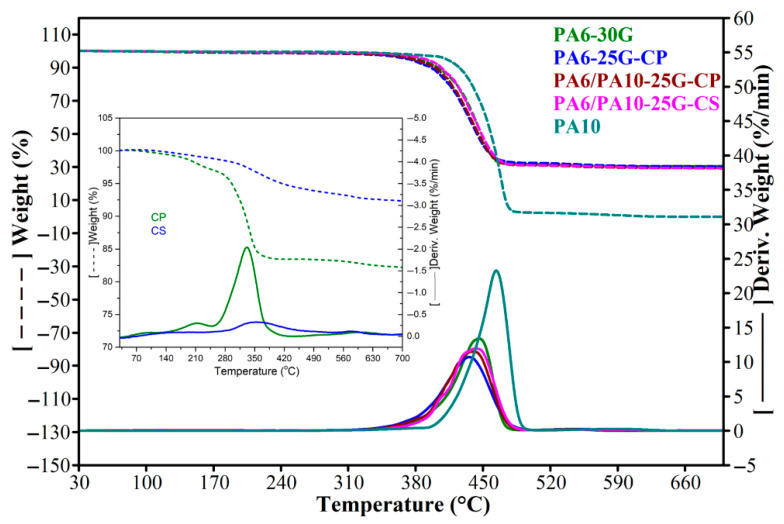
TGA and DTGA curves of PA10, PA6-30G, PA6-25G-CP, PA6/PA10-25G-CP, and PA6/PA10-25G-CS composites.

A single weight-loss step was observed according to the TGA data (Figure 5 and Table 5) for all samples between 300 and 500 °C. For hygroscopic materials, weight loss typically occurs between 50 and 200 °C due to water or moisture evaporation. In our case and similar to a study performed by Sheikh et al. [62], GF-reinforced PA6 samples exhibit the burning off of low-molecular-weight species below 200 °C, amounting to around 1–2% (as seen in Table 5), possibly because of moisture evaporation. From Table 5, it can be seen that, up to 230 °C, the PA6 and PA6/PA10 composites lose 17–52% less weight than PA6-30G, indicating better thermal stability at the processing temperature of the obtained composites. Above 400 °C, the thermal stability of PA6 and PA6/PA10 composites is lower than PA6-30G. The onset temperature and the temperature at the maximum rate of decomposition (T_max_) decrease by 7–14 °C and 8–10 °C, respectively. The PA6-25G-CP composite has the lowest thermal stability. By adding PA10 in PA10/PA6-25G-CP composite, the thermal stability improves (the onset temperature, T_max_, and the temperature at which the weight loss is 5% increase by 1.6, 2.8, and 4 °C). Compared to the PA6 and PA6/PA10 composites, PA10 has the highest thermal stability based on the value of T_max_, which is +20 °C more compared to PA6-30G and almost +30 °C compared to PA6/PA10-25G-CP and PA6/PA10-25G-CS. Even though PA6-25G-CP has a lower T_max_ value compared to PA6-30G, the introduction of PA10 increases the T_max_ values for PA6/PA10-25G-CP and PA6/PA10-25G-CS, highlighting the positive effect of PA10 on thermal stability. Treating the ash with silane has the effect of improving the thermal stability of the ash [25] and, further, of the PA6/PA10-25G-CS composite (the onset temperature increases by approximately 5 °C, and the temperature at which the loss is 5% increases by approximately 3 °C, compared to the PA6/PA10-25G-CP composite). This improved thermal stability is the evidence of a higher degree of interaction between the components.

Composite PA6-25G-CP has lower thermal stability compared to PA6-30G based on the T_max_ value in Table 5. This decrease of approx. 10 °C may be caused by several factors. Firstly, the fiber content reduced by 5% might play a key role in this decrease in thermal stability as GF tend to increase the thermal stability of polymer composites due to their improved heat transfer characteristics [63]. Secondly, the surface treated ash powder may also play an important role in this decrease, since, according to the data in Table 5, CP has a lower thermal stability compared to all other samples. Its degradation process starts earlier than the other samples based on the onset point at 299.4 °C. The temperature at which it loses 5% weight and the T_max_ are much lower compared to all other samples. The thermal decomposition behavior for PA6/PA10-25G-CP and PA6/PA10-25G-CS is more similar to that of PA6-30G than PA6-25G-CP, indicating strong interactions between the components and good dispersion of treated ash in the polymer hybrid matrix.

The TGA results are consistent with the FTIR, DSC, and XRD results.

### 3.5. Mechanical and Dynamic Mechanical Analysis

**Figure 6:**
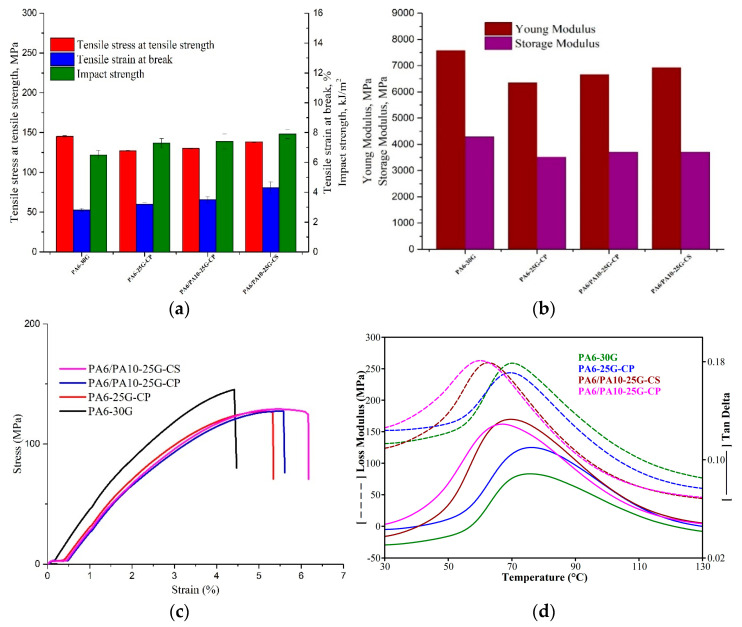
Mechanical and dynamic mechanical properties of PA6-30G, PA6-25G-CP, PA6/PA10-25G-CP, and PA6/PA10-25G-CS composites (**a**). Tensile and impact properties. (**b**) Young modulus and storage modulus. (**c**) Stress vs. strain curves. (**d**) Loss modulus and loss factors.

GFs are used as a reinforcing agent for many polymer products to form a very strong and relatively light composite material. For many automotive applications, a balance of mechanical properties is required because the large increase in one property may result in the downgrading of another property. For reinforced thermoplastic composites, the most important properties are high toughness, represented by tensile strain and impact strength, along with high stiffness and strength. Considering Figure 6a, compared to PA6-30G, the tensile strength of PA6-25G-CP decreases by 12%, while for PA6/PA10-25G-CP and PA6/PA10-25G-CS, the decrease is about 10% and 5%, respectively. Despite this decrease in tensile strength and material rigidity seen in Figure 6a,b, where the Young modulus and storage modulus of PA6-25G-CP and PA6/PA10 composites are lower than that of PA6-30G by approx. 8–18%, we notice, in Figure 6a, an increase in tensile strain and impact strength for the ash-treated composites compared to neat PA6-30G. In the case of automotive parts, such as bumpers, which play an essential role in protecting passengers and vehicles during a collision, the properties of the composite materials used in their manufacture are particularly important. In addition to high specific stiffness and strength, impact resistance and flexibility are also very important. During a collision, the part must simultaneously have strength and stiffness to limit deformation, but also sufficient elastic deformability to absorb and dissipate the impact energy. According to scientific data [14,64,65], a decrease in rigidity and tensile strength for a composite may provide better results in applications that require enhanced energy absorption and impact strength. This approach is already put in practice in the automotive industry by replacing metallic automotive components that have high rigidity with polymer composites with lower rigidity but improved impact strength and elasticity. Examples of car parts that underwent this kind of transition include car bumpers and door panels. Studies by various researchers [66] have shown that, during a collision, a bumper made of a composite material with high strength and stiffness exhibits low deformation, but also a lower transfer of velocity and kinetic energy between the bumper and the vehicle. Therefore, it is important to have a good balance between properties and in the case of PA6/PA10 composites a decrease in stiffness and strength by up to 15%, but an increase in elastic deformability by 25–54% and impact resistance by 11–18% should not affect the performance of automotive parts.

The highest values for tensile strain and impact strength can be seen for PA6/PA10-25G-CS, with an increase of approx. 35% and 18%, respectively, compared to PA6-30G. Even with this increase in tensile strain, all specimens broke in a brittle manner, as seen from the stress vs. strain curves in Figure 6c. The improvement in both tensile strain and impact strength for PA6/PA10-25G-CP and PA6/PA10-25G-CS is mainly due to the presence of PA10. This techno-polymer has much higher values for tensile strain and impact strength according to its data sheet. The hybrid PA6/PA10 composites present improved toughness properties compared to PA6-30G, confirming the good compatibility between the two polymers that we saw in DSC analysis from the melting peaks. At the same time, these hybrid composites also show higher values for stiffness and mechanical strength compared to the PA6-25G-CP composite, which is evidence of a stronger interaction at the polymer matrix–reinforcement-agent interface.

Compared to the PA6-25G-CP and PA6/PA10-25G-CP composites, the PA6/PA10-25G-CS composite presented the highest values for both stiffness and toughness (Table 6) due to the effect of the silane surface treatment of the fly ash. This behavior was also confirmed by Wang et al. [67], who showed that the introduction of fly ash treated with aluminum silicate and a silane coupling agent into pure PA6 led to an increase in the impact strength, elastic modulus, and tensile strength of the obtained composites.

Loss modulus and tan δ curves for the PA6 and PA6/PA10 composites are represented in Figure 6d. One transition region is recorded in the temperature range of 30–130 °C for all samples, which is associated with the α-transition, and the highest peak of the loss modulus curve or the maximum of tan δ peak are generally associated with glass transition temperature (T_g_) of PA6 [68]. There is a significant difference in the magnitude of tan δ between PA6-30G and the other composites, as seen in Figure 6d and Table 7. Composites PA6-25G-CP, PA6/PA10-25G-CP, and PA6/PA10-25G-CS have higher tan δ peaks, an indication of their improved toughness. The PA6/PA10-25G-CS composite recorded the highest peak value, which correlates very well with the highest values for tensile strain at break and impact strength obtained for this composite. This composite also presents the lowest values for T_g_ which indicates a high flexibility compared to PA6-30G composite. Studies by López-Manchado et al. [69] and by Hassan et al. [70] have shown that a higher concentration of GF may act as barrier to the polymer chain’s mobility, being responsible for a reduction in the degree of molecular motion and, in turn, the damping properties, as well as the fact that there would be less matrix by volume to disperse the vibration energy. We notice a decrease in T_g_ to values of 60.1 °C and 62.76 °C for PA6/PA10-25G-CP and PA6/PA10-25G-CS compared to PA6-30G (70.16 °C). This behavior indicates an increase in polymer chain mobility, on the one hand due to PA10 as an effect of weakening the hydrogen bond network, and on the other hand due to the plasticizer effect of the fly ash surface treatment agents. A similar behavior was also reported by Quiles-Carrillo et al. [71], who attributed the decrease in T_g_ of the PA10 to the plasticizing effect of the compatibilizers of the PA10/polylactic acid blend.

### 3.6. SEM Analysis

**Figure 7:**
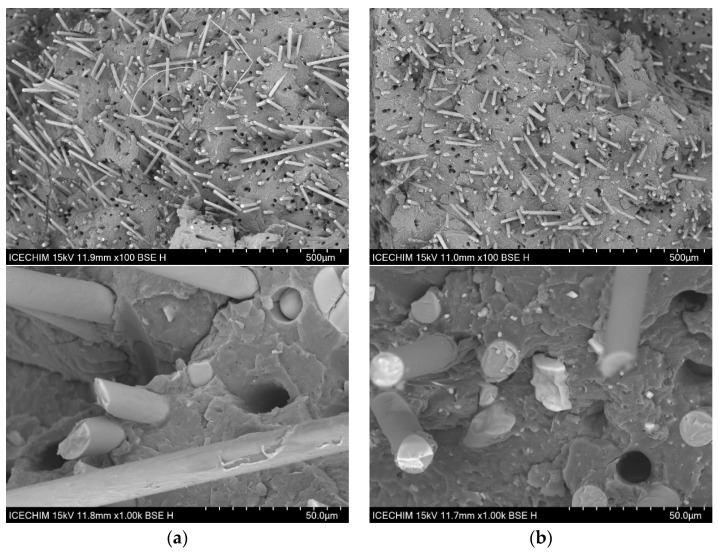

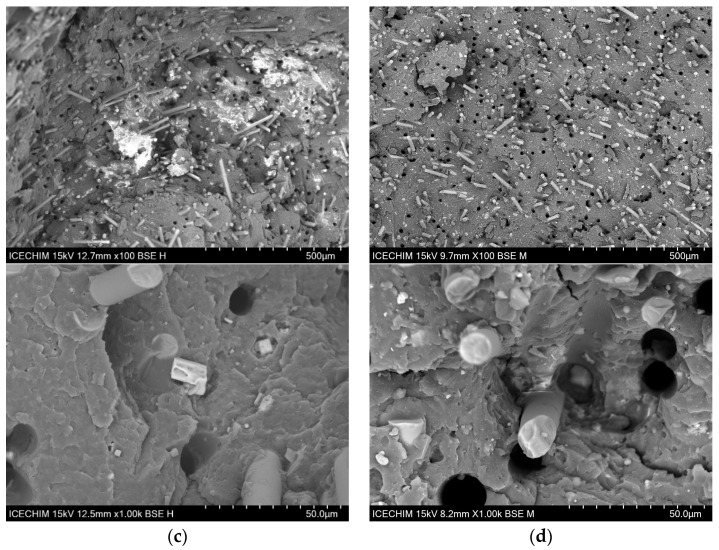
SEM surface morphology (100×) and (1000×) of (**a**) PA6-30G; (**b**) PA6-25G-CP composite; (**c**) PA6/PA10-25G-CP composite; (**d**) PA6/PA10-25G-CS composite.

Figure 7a displays the morphology of PP-30G, highlighting the GF embedded in the polymer matrix. Uniformly distributed holes are evident in each sample, indicating that the GF are evenly dispersed within the polymer matrix. The surface of the GF displays residual material from the polymer matrix after fracturing, demonstrating strong adhesion between the fiber and the matrix. Research by Thomason et al. [72] and Fu et al. [73] suggests that the amount of polymer material still attached to the fibers can reveal the quality of fiber–matrix adhesion. Additionally, the non-perfectly round shapes of the holes left by the removed GF suggest that organic material remains attached to the fibers when they are pulled out. As ash powder is added, smaller- and larger-sized agglomerates can be seen in Figure 7b–d. It is important to highlight that the smaller ash particles are firmly embedded in the polymer matrix, since there is no separation visible, and the pullout effect is lessened when compared to PP-30G (both in terms of the irregular form of the holes and the lower percentage of holes). Usually, when mixing two different polymers, issues regarding the interfacial adhesion between them are raised. Books written by Robenson [74] and Isayev [75] discuss in detail how the stability between immiscible polymers can be obtained through the use of compatibilizing agents that reduce the interfacial tension between the two polymer phases. In our case, the blend of polar polymers (PA6 and PA10) did not result in visible phase separation. Figure 7c,d shows a good mixture between the polymers with the GF and ash particles well-dispersed in the polymer material. If phase separation would have occurred, it would have been expected for us to observe various droplets of PA10 within PA6-30G and an unstable morphology that reduces the total free energy of the system. In a study performed by Laoutid et al. [76], the incorporation of 5% hydrophilic nanosilica resulted in a much finer dispersion of polyamide and polycarbonate within a polypropylene matrix due to the preferential accumulation of silica nanoparticles at the interface. In our case, it is unclear whether the ash particles influence the interface between the PA6 and PA10 phases. However, as pointed out during the DSC analysis, the shift in the melting curves indicates a strong interaction between the polymers. Larger cubic structures can be seen in several images in Figure 7, at 1000×, that are associated with the halite and sylvite components from the ash powder. These large structures are few and do not seem to be entirely separated from the polymer matrix; however, the smaller structures and particles are clearly well dispersed within the matrix.

### 3.7. Aspects Regarding Recyclability and Industrial Feasibility

In terms of recyclability and industrial feasibility, the hybrid composites analyzed in this study perform as well or even better than conventional PA6-GF systems, for several reasons. The conventional systems withstand 3–5 recycling cycles before critical degradation [73], with a 20–30% reduction in impact strength after the first cycle of recycling and a decrease by 50% of the glass-fiber aspect ratio after reprocessing [77]. Compared to conventional systems, PA6/PA10 composites could withstand an equal or even higher number of recycling cycles, considering the presence of treated fly ash and PA10, which improve the degradation resistance of the composite. This behavior should be reflected in the prevention of polymer chain scission and the preservation of mechanical strength in proportion of 85–90%. The fly ash surface treatment agent also has a lubricating effect, reducing fiber breakage and improving melt-flow (lower energy consumption and costs) [75,76]. Composites with lower glass-fiber content will be easier to recycle than composites with higher glass-fiber content, and will have similar mechanical strengths. At the same time, the problems related to the presence of glass fiber in the recycled material melt (dispersion, polymer melt-flow, and wear of recycling equipment) will be reduced. These observations are speculative, and further investigations are required in future analyses, as well as investigations regarding the environmental contamination or health risks associated with our composites.

## 4. Conclusions

Hybrid composites comprising polyamide 6 (PA6) reinforced with glass fibers (GF), supplemented with treated thermal power plant fly ash and bio-based polyamide 10.10 (PA10), were obtained via melt processing under dynamic conditions. This study systematically investigates the combined effects of these components on the structural, morphological, thermal, and mechanical properties of the GF-reinforced PA6 matrix. Comprehensive analyses were conducted to elucidate the nature and extent of interfacial interactions within the composite system. Morpho-structural analysis revealed strong interfacial interactions among constituents, with no observable phase separation between PA6 and PA10. Treated fly ash particles exhibited a homogeneous dispersion throughout the polymer matrix. Thermal analysis indicated minimal variation for the secondary melting temperature (Tm2) with the incorporation of PA10 and treated fly ash, accompanied by a slight decrease in melting enthalpy and crystallinity relative to the PA6-25G-CP composite. Notably, PA10 enhanced the thermal stability of the hybrid composites, evidenced by increases in both the temperature at the maximum rate of decomposition (by approx. 2–3 °C) and the temperature at 5% mass loss (by approx. 4–14 °C). Mechanically, the inclusion of PA10 in PA6-25G-CP significantly enhanced the composite’s toughness, evidenced as an 18% and 35% increases in impact strength and tensile strain at break, respectively. It is important to note that the improvement in impact strength and tensile strain in polymer composites is usually achieved with the incorporation of a thermoplastic elastomer; however, in this case, the increase was achieved by using less than 10% PA10. This improvement occurred at the same time as an 8–18% reduction in stiffness for the hybrid composites compared to PA6-30G. Comparative analysis demonstrated that the PA6/PA10-25G-CS composite, incorporating silane-treated fly ash, achieved optimal balance of properties, exhibiting higher stiffness and toughness compared to both PA6-25G-CP and PA6/PA10-25G-CP composites. This enhancement is attributed to improved interfacial adhesion resulting from the silane surface modification of fly ash. Consequently, these results demonstrate a viable pathway for developing sustainable alternatives to conventional GF-reinforced PA6. The partial replacement of glass fibers with surface-modified fly ash and the incorporation of PA10 led to the achievement of composites (particularly PA6/PA10-25G-CS) that meet the key property requirements specific for automotive applications. The significant toughness enhancement positions this composite as particularly suitable for impact-critical components, such as bumpers or door panels, where energy absorption is paramount. Further investigations will also investigate the recyclability and industrial feasibility of the obtained hybrid composites.

## Figures and Tables

**Table 1:** The PA6 and PA6/PA10 hybrid composite formulations.

Sample	PA6 (wt.%)	GF (wt.%)	PA10 (wt.%)	CP (wt.%)	CS (wt.%)
PA6-30G	70	30	-	-	-
PA6-25G-CP	68.5	25	-	6.5	-
PA6/PA10-25G-CP	59.8	24.5	9.2	6.5	-
PA6/PA10-25G-CS	59.85	24.6	9.8	-	5.75

**Table 2:** Assignments of the FTIR bands for PA6 and PA6/PA10 composites.

PA6-30G, cm^−1^	PA6-25GF-CP, cm^−1^	PA6/PA10-25GF-CP, cm^−1^	PA6/PA10-25GF-CS, cm^−1^	Band Assignment	
3298	3298	3300	3300	N–H stretching vibration	General
3075	3075	3075	3075	N–H stretch and amide II overtone	General
2922	2922	2919	2919	CH_2_ asymmetric stretching	General
2855	2855	2850	2850	CH_2_ symmetric stretching	General
1633	1633	1635	1635	Amide I band (C=O stretching)	General
1537	1537	1539	1539	Amide II band (N–H in-plane bending coupled with C–N and C–O stretch)	General
1462	1462	1467	1467	CH_2_ scissoring not adjacent to the amide group	α-structure
1435	1435	1435	1435	CH_2_ scissors vibration	γ-structure
1420	1420	1420	1420	CH_2_ scissoring	α-structure
1236	1236	1237	1237	CH_2_ twist-wagging	γ-structure
725	725	726	726	Rocking mode of CH_2_	γ-structure

**Table 3:** DSC results for PA6-30G, PA6-25G-CP, PA6/PA10-25G-CP, and PA6/PA10-25G-CS composites.

Ramp 10 °C/min	Crystallization				Melting 1 (M1)			Melting 2 (M2)			Melting Total (M1 + M2)	
	Onset	T_c_	ΔH_c_	X_c_	Onset	T_m1_	ΔH_m1_	Onset	T_m2_	ΔH_m2_	ΔH_m_	X_c_
(He, 25 mL/min)	°C	°C	J/(g)	%	°C	°C	J/(g)	°C	°C	J/(g)	J/(g)	%
PA6-30G	205.1	196.9	41.68	25.9	-	-	-	207.2	217.9	48.32	48.32	30.0
PA6-25G-CP	199.6	191.3	41.04	26.0	-	-	-	200.4	214.2	45.73	45.73	29.0
PA6/PA10-25G-CP	198.2	190.5	41.32	30.0	176.1	198.6	17.46	201.3	214.2	27.80	45.26	28.5
PA6/PA10-25G-CS	196.9	189.3	40.21	29.2	181.3	197.5	16.65	201.9	214.5	26.58	43.23	27.0

**Table 4:** XRD results for the studied PA6 and PA6/PA10 composites.

		αPA6.6/PA10 (200)	αPA6.6/PA10 (002) + (202)
PA6-30G CI = 73%	2θ (°)	21.04	22.94
	Height (cps)	5715	8033
	FWHM (°)	2.84	2.53
PA6-25G-CP CI = 57%	2θ (°)	21.22	22.62
	Height (cps)	5146	7761
	FWHM (°)	2.49	2.48
PA6/PA10-25G-CP CI = 53%	2θ (°)	20.97	22.90
	Height (cps)	4971	4360
	FWHM (°)	3.05	2.34
PA6/PA10-25G-CS CI = 67%	2θ (°)	20.78	22.68
	Height (cps)	6053	8393
	FWHM (°)	2.73	2.77

**Table 5:** TGA results of PA10, PA6-30G, PA6-25G-CP, PA6/PA10-25G-CP, and PA6/PA10-25G-CS composites.

Sample	RT—230 °C Wt. Loss %	Onset Point Temp °C	T_max_ °C	Residue 700 °C	Temp for Wt. Loss 5% °C
PA10	0.39	440.8	468.0	0.20	412.0
CP	3.63	299.4	336.7	74.28	267.4
CS	0.97	298.1	350.0	92.35	415.0
PA6-30G	1.49	416.1	448.6	30.78	382.0
PA6-25G-CP	1.23	402.1	438.2	30.26	374.0
PA6/PA10-25G-CP	1.11	403.7	441.0	29.22	378.0
PA6/PA10-25G-CS	0.71	408.9	440.0	29.15	390.6

**Table 6:** Mechanical properties of PA6-30G, PA6-25G-CP, PA6/PA10-25G-CP, and PA6/PA10-25G-CS composites.

Sample	Tensile Stress at Tensile Strength (MPa)	Young’s Modulus (MPa)	Tensile Strain at Break (%)	Impact Strength (KJ/m^2^)
PA6-30G	145.1 ± 1	7578 ± 70	2.8 ± 0.1	6.5 ± 0.3
PA6-25G-CP	127 ± 0.4	6347 ± 91	3.2 ± 0.1	7.3 ± 0.3
PA6/PA10-25G-CP	130 ± 0.4	6664 ± 78	3.5 ± 0.2	7.4 ± 0.5
PA6/PA10-25G-CS	138 ± 0.4	6930 ± 37	4.3 ± 0.4	7.9 ± 0.3

**Table 7:** Storage modulus, loss modulus, and loss factor values for PA6 and PA6/PA10 composites.

Sample	Storage Modulus, E′		Loss Modulus, E″		Loss Factor	
	Temp	E′	Temp	E″ Peak	Temp	Tan Delta
	°C	MPa	°C	MPa	°C	Tan δ Peak
PA6-30G	30	4287	70.2	258.7	75.76	0.089
PA6-25GF-CP	30	3518	69.5	243.6	76.23	0.110
PA6/PA10-25GF-CP	30	3702	60.1	262.7	67.01	0.129
PA6/PA10-25GF-CS	30	3733	62.8	259.5	69.74	0.133

## Data Availability

Data are contained within the article.
